# Supplementary material for: The prevalence of anxiety and related factors among primary and secondary school teachers in Hanoi, Vietnam, during the COVID-19 pandemic in 2020
Source: PLOS Glob Public Health. 2024 Feb 28;4(2):e0002932. doi: 10.1371/journal.pgph.0002932 (PMC10901312; doi:10.1371/journal.pgph.0002932)
Supplement: Attachment — Submitted filename: Reviewers Comments PLOS.docx [file pgph.0002932.s003.docx]

**Reviewer’s Comments PLOS**

**Title**:

I suggest the title should read “…among primary and secondary school teachers in Hanoi…,” rather than “…in among primary and secondary school teachers in Hanoi…”

**Abstract**

1. According to the journal guidelines, the abstract should not have distinct subheadings.
2. The background section of the abstract started off with what could best be a conclusion. If Anxiety was already established to be prevalent in the population, why study the prevalence again? I suggest the author(s) start with either a problem statement or they should go straight to the major purpose of the study.
3. Line 34 – The authors should correct the grammar in this sentence; “Teachers with intention to change job **had** 3.30 times more likely to…” the highlighted word should be changed to ‘were’.

**Introduction**:

Page 4: The information in lines 73 -77 is more appropriate in the method section (it was repeated in the methods section anyways).

The authors listed many factors which they anticipated would be associated with teachers’ anxiety, but there was theoretical justification for assessing the independent variables mentioned. I suggest the authors frame the study with one or two theories.

**Methods**

The authors need to give the actual number, rather than stating “several” schools from where they selected the teachers. The population of the teachers in those districts is also needed to justify the rather small sample size, considering that both primary and secondary school teachers participated.

Line 101: I suppose the word is “mild” and not “mid”.

The authors stated that “DASS-42 has been validated among Vietnamese community”, but I wonder if the subscale of “Anxiety” has been validated in isolation of the other subscales (Depression and Stress) in that population? The authors need to justify that with a reliability test result.

The authors should place texts under the correct subheadings to avoid repeating information. For instance; the instrument for assessing anxiety was described under study variables whereas there was a subheading for Data collection tools. I suggest the authors merge the information that relates to study variables with the information on data collection tools.

Alternatively, under study variables, they could focus on describing the dependent variable and the independent variables. They should then have a separate section for data collection tools where they explain the DASS-42 scale and the questionnaire they used to assess the independent variables. They also need to give a sample item of the DASS-42 for assessing anxiety.

The information on how anxiety was categorized should be taken to the Data analysis section.

Obtaining consent forms from the participants was stated twice; in the data collection section and ethical approval section. The authors should have the information in one section.

Line 148 &149: it is not proper to start a sentence with a figure.

Line 149: the authors wrote; “…average income <10 million”. The authors need to clarify whether it was monthly or annual income. They also need to add the currency symbol.

The tables’ contents should have uniform decimal places.

**Discussion**

The authors made several comparisons of their prevalence finding with the finding of previous studies, with minimal interpretation of their own findings. I suggest the authors further explain the implications of such a high prevalence of anxiety in the population under study.

It is not clear what the authors mean by repetitive work and how it could increase the risk of anxiety. Framing all these concepts theoretically would make the discussion more meaningful.

**Conclusion**

The conclusion is still very much underdeveloped. I believe that if the authors properly justify the reason they assessed the various independent variables, they might come up with a more robust discussion and conclusion. (For instance, if the variables were assessed based on the conflicting findings of previous studies, or in the advancement of a psychological theory, the authors can report and discuss the variables that were not significant and give the implications of such findings to the Vietnamese teachers).
