# Supplementary material for: The prevalence of anxiety and related factors among primary and secondary school teachers in Hanoi, Vietnam, during the COVID-19 pandemic in 2020
Source: PLOS Glob Public Health. 2024 Feb 28;4(2):e0002932. doi: 10.1371/journal.pgph.0002932 (PMC10901312; doi:10.1371/journal.pgph.0002932)
Supplement: Attachment — Submitted filename: PGPH-D-23-00934 Reviewers Comments PLOS.docx [file pgph.0002932.s004.docx]

PGPH-D-23-00934
The prevalence of anxiety and related factors in primary and secondary school teachers in Hanoi, Vietnam during COVID-19 pandemic in 2020
PLOS Global Public Health

RESPONSE TO REVIEWER

Journal Requirements:

1. PLOS requires corresponding authors to have an ORCID iD. Please ensure that you have an ORCID iD and that it is validated in Editorial Manager system. To do this, go to ‘Update my Information’ (in the upper left-hand corner of the Editorial Manager main menu), and click on the Fetch/Validate link next to the ORCID field. This will take you to the ORCID site and allow you to create a new iD or authenticate a pre-existing iD in Editorial Manager. For more information please visit: <https://www.plos.org/orcid>.

Response:

Thank you very much for your comment. The corresponding author updated their ORCID ID in the journal system.

2. Please amend your online Financial Disclosure statement. If you did not receive any funding for this study, please simply state: “The authors received no specific funding for this work.”

Response:

Thank the reviewer very much for your comment. The author stated, “The authors received no specific funding for this work.”

3. Please update your online Competing Interests statement. If you have no competing interests to declare, please state: “The authors have declared that no competing interests exist.”

Response:

Thank the reviewer very much for your comment. The author stated, “The authors have declared that no competing interests exist.”

4. In the online submission form, you indicated that "Data is available upon request to the corresponding author.". All PLOS journals now require all data underlying the findings described in their manuscript to be freely available to other researchers, either 1. In a public repository, 2. Within the manuscript itself, or 3. Uploaded as supplementary information.
This policy applies to all data except where public deposition would breach compliance with the protocol approved by your research ethics board. If your data cannot be made publicly available for ethical or legal reasons (e.g., public availability would compromise patient privacy), please explain your reasons by return email and your exemption request will be escalated to the editor for approval. Your exemption request will be handled independently and will not hold up the peer review process, but will need to be resolved should your manuscript be accepted for publication. One of the Editorial team will then be in touch if there are any issues.

Response:

Thank the reviewer very much for your comment. The data was uploaded as supplementary information.

5. Please ensure that you cite or refer to Table 1 in your text as, if accepted, production will need this reference to link the reader to the table.

Response:

Thank the reviewer very much for your comment. Table 1 was cited in the text.

**Reviewer’s Comments PLOS**

**Title**:

I suggest the title should read “…among primary and secondary school teachers in Hanoi…,” rather than “…in among primary and secondary school teachers in Hanoi…”

Response:

Thank the reviewer very much for your comment. The title was revised accordingly as follows:

**“The prevalence of anxiety and related factors among primary and secondary school teachers in Hanoi, Vietnam, during the COVID-19 pandemic in 2020”**

**Abstract**

1. According to the journal guidelines, the abstract should not have distinct subheadings.
2. The background section of the abstract started off with what could best be a conclusion. If Anxiety was already established to be prevalent in the population, why study the prevalence again? I suggest the author(s) start with either a problem statement or they should go straight to the major purpose of the study.
3. Line 34 – The authors should correct the grammar in this sentence; “Teachers with intention to change job **had** 3.30 times more likely to…” the highlighted word should be changed to ‘were’.

Response:

Thank the reviewer very much for your comment. Subheadings were removed. The abstract was revised. Grammar errors were fixed.

**Introduction**:

Page 4: The information in lines 73 -77 is more appropriate in the method section (it was repeated in the methods section anyways).

Response:

Thank the reviewer very much for your comment. We removed this sentence from data collection in the Introduction and kept it in the Method section.

The authors listed many factors which they anticipated would be associated with teachers’ anxiety, but there was theoretical justification for assessing the independent variables mentioned. I suggest the authors frame the study with one or two theories.

Response:

Thank the reviewer very much for your comment. The independent variables were selected based on the literature review. In the introduction, we also summarized the factors associated with teachers’ anxiety from previous studies. This paper examined these associations among Vietnamese teachers.

**Methods**

The authors need to give the actual number, rather than stating “several” schools from where they selected the teachers. The population of the teachers in those districts is also needed to justify the rather small sample size, considering that both primary and secondary school teachers participated.

Response:

Thank the reviewer very much for your comment. In the abstract, study participants, and sample size, we stated that the participants were from 10 primary and secondary schools. This paper analyzed data collected by another study, so it is impossible for us to do anything about sample size and selection. We added some sentences in the limitation section to address this matter.

Line 101: I suppose the word is “mild” and not “mid”.

Response:

Thank the reviewer very much for your comment. The spelling mistake was revised.

The authors stated that “DASS-42 has been validated among Vietnamese community”, but I wonder if the subscale of “Anxiety” has been validated in isolation of the other subscales (Depression and Stress) in that population? The authors need to justify that with a reliability test result.

Response:

Thank the reviewer very much for your comment. We supplemented information on Anxiety reliability in terms of Cronbach alpha’s coefficient, which was satisfactory (0.89).

The authors should place texts under the correct subheadings to avoid repeating information. For instance, the instrument for assessing anxiety was described under study variables whereas there was a subheading for Data collection tools. I suggest the authors merge the information that relates to study variables with the information on data collection tools.

Response:

Thank the reviewer very much for your comment. We merged sections Variables and Data collection tools appropriately to avoid duplication.

Alternatively, under study variables, they could focus on describing the dependent variable and the independent variables. They should then have a separate section for data collection tools where they explain the DASS-42 scale and the questionnaire they used to assess the independent variables. They also need to give a sample item of the DASS-42 for assessing anxiety.

Response:

Thank the reviewer very much for your comment. We merged sections Variables and Data collection tools appropriately to avoid duplication. Samples of the DASS-42 anxiety subscale were provided.

The information on how anxiety was categorized should be taken to the Data analysis section.

Response:

Thank the reviewer very much for your comment. Categorization of Anxiety score was provided in the Variable section, where we described the dependent variable (Anxiety)

Obtaining consent forms from the participants was stated twice, in the data collection section and ethical approval section. The authors should have the information in one section.

Response:

Thank the reviewer very much for your comment. We revised and put the consent information in the data collection section since it was part of a data collection procedure.

Line 148 &149: it is not proper to start a sentence with a figure.

Response:

Thank the reviewer very much for your comment. We revised the sentences.

Line 149: the authors wrote; “…average income <10 million”. The authors need to clarify whether it was monthly or annual income. They also need to add the currency symbol.

Response:

Thank the reviewer very much for your comment. The number was the average monthly income, the currency was VND. We revised the sentence.

The tables’ contents should have uniform decimal places.

Response:

Thank the reviewer very much for your comment. we revised the number in the table and kept one decimal place in the table

**Discussion**

The authors made several comparisons of their prevalence finding with the finding of previous studies, with minimal interpretation of their own findings. I suggest the authors further explain the implications of such a high prevalence of anxiety in the population under study.

Response:

Thank the reviewer very much for your comment. We provided implications on the high prevalence of anxiety.

It is not clear what the authors mean by repetitive work and how it could increase the risk of anxiety. Framing all these concepts theoretically would make the discussion more meaningful.

Response:

Thank the reviewer very much for your comment. We provided additional information on “repetitive work” and its impact on anxiety.

**Conclusion**

The conclusion is still very much underdeveloped. I believe that if the authors properly justify the reason they assessed the various independent variables, they might come up with a more robust discussion and conclusion. (For instance, if the variables were assessed based on the conflicting findings of previous studies, or in the advancement of a psychological theory, the authors can report and discuss the variables that were not significant and give the implications of such findings to the Vietnamese teachers).

Response:

Thank reviewer very much for your comment. We revised the discussion and conclusion in more detail.
