# Supplementary material for: The prevalence of anxiety and related factors among primary and secondary school teachers in Hanoi, Vietnam, during the COVID-19 pandemic in 2020
Source: PLOS Glob Public Health. 2024 Feb 28;4(2):e0002932. doi: 10.1371/journal.pgph.0002932 (PMC10901312; doi:10.1371/journal.pgph.0002932)
Supplement: Attachment — Submitted filename: Reviewers comments to the author 2.docx [file pgph.0002932.s005.docx]

**Reviewer’s comments to the author(s)**

The author(s) have made a remarkable revision of their manuscript. It is currently much better than the previous copy. Here are a few more corrections to improve the work.

Page 5; Lines 110-111

The authors have added the Cronbach alpha value (0.89) to show the reliability of the anxiety subscale. I suggest the authors add a text describing what the alpha value indicates. Example. “the DASS-42 Anxiety Cronbach alpha’s was 0.89 which indicates high internal consistency of the test items.”

Page 6;

Line 127: The text should read better as “students’ safety”

Line 128: use “non-academic work” instead of “non-education work”. Correct also in the table.

Line 130: It is not clear what the authors mean by the text “inappropriate diet/rest schedule”. It appears “diet” is synonymous with “rest” and again the word “diet” does not seem proper in the context. The authors may consider recasting the sentence as “inadequate eating and resting schedule at school” (if this captures what they intend to convey).

The phrase “unsatisfied remuneration regime” should better read as “unsatisfactory remuneration regime”

Page 7

Line 141: the word is “**administered”**  and not “administrated”

Line 144: The text should read “the data were” not “the data was”

Line 156: the text “ Decriptive data were presented” seems incomplete. Authors should describe how the data were presented.

Page 8

Line 170: The text should read “The final analysis of data” not “The final analysis of this paper”.

Line 173: The figure in bracket (85.7%) should come immediately after the word “most’ (Most [85.7%] respondents…)

Page 9

Line 178: should read “prevalence of anxiety symptoms among the participants” not “prevalence of participants…”

Page 10

Table 2: it is not clear why the percentage figures presented, do not sum up to 100. Again, the authors did not indicate anywhere in the manuscript that the analysis presented involved only the participants who showed anxiety symptoms.

The authors need to also correct “Be criticized” and “Be disciplined” in the table.

Page 14

Line 253: The text should read “Parent-**teacher** collaboration plays a significant role in **students’** learning…”

Page 15

The authors have explanations for the high prevalence of anxiety in Malasia and Egypt, but has very little to say about the possible causes of the high prevavalence of anxiety in Vietnam. I suggest the authors discuss their findings more. “What might be the possible cause/causes of teachers’ anxiety? What are the likely consequences of teachers’ anxiety on the individual teachers, school children, and Vietnam society at large.

Lines 273-274: The authors have still not discussed the finding on the relationship between teachers’ anxiety and intention to leave. The authors simply wrote that “a significant association was found between anxiety and intention to leave.” But nothing was said about the direction of the associaton. I expect the authors to further explain that teachers who had the intention to quit the job showed higher odds of anxiety symptoms than those who had no intention to leave the job.

Again, the authors wrote that their “finding imply that psychological interventions improve teachers’ mental health and help them stay with their current job.” However, I do not think that is the major implication of that finding. I think a major implication should be that “when teachers experience increased fear and worry about their work situations, they might lack the enthusiasm for the work and therefore, might want to quit the job. Conversely, teachers who already intend to leave the job, might experience increased anxiety as they handle the strain and stress associated with teaching young people.”

Page 16

Lines 275 and 276: The text is a recommendation for future study. It should be taken to the last part of the conclusion section.

I believe these additional improvements will be helpful to the authors.
