# Supplementary material for: The prevalence of anxiety and related factors among primary and secondary school teachers in Hanoi, Vietnam, during the COVID-19 pandemic in 2020
Source: PLOS Glob Public Health. 2024 Feb 28;4(2):e0002932. doi: 10.1371/journal.pgph.0002932 (PMC10901312; doi:10.1371/journal.pgph.0002932)
Supplement: Attachment — Submitted filename: Reviewers comments to the editor2.docx [file pgph.0002932.s006.docx]

**Reviewer’s comments to the editor**

Thank you for giving me another opportunity to review the revised copy of the manuscript. The authors have improved the manuscript, but the discussion section still lacks sufficient discussion of their findings.

I, therefore, recommend a moderate revision
