# Supplementary material for: The prevalence of anxiety and related factors among primary and secondary school teachers in Hanoi, Vietnam, during the COVID-19 pandemic in 2020
Source: PLOS Glob Public Health. 2024 Feb 28;4(2):e0002932. doi: 10.1371/journal.pgph.0002932 (PMC10901312; doi:10.1371/journal.pgph.0002932)
Supplement: Attachment — Submitted filename: PGPH-D-23-00934 Reviewers Comments PLOS _2nd.docx [file pgph.0002932.s007.docx]

PGPH-D-23-00934
The prevalence of anxiety and related factors in primary and secondary school teachers in Hanoi, Vietnam during COVID-19 pandemic in 2020
PLOS Global Public Health

RESPONSE TO REVIEWER

| reviewer 1: |  |
| --- | --- |
| The author(s) have made a remarkable revision of their manuscript. It is currently much better than the previous copy. Here are a few more corrections to improve the work.  Page 5; Lines 110-111  The authors have added the Cronbach alpha value (0.89) to show the reliability of the anxiety subscale. I suggest the authors add a text describing what the alpha value indicates. Example. “the DASS-42 Anxiety Cronbach alpha’s was 0.89 which indicates high internal consistency of the test items.” | Thank you very much for your comment, we revised the part as you suggested by adding the meaning of Cronbach alpha. |
| Page 6;  Line 127: The text should read better as “students’ safety”  Line 128: use “non-academic work” instead of “non-education work”. Correct also in the table.  Line 130: It is not clear what the authors mean by the text “inappropriate diet/rest schedule”. It appears “diet” is synonymous with “rest” and again the word “diet” does not seem proper in the context. The authors may consider recasting the sentence as “inadequate eating and resting schedule at school” (if this captures what they intend to convey).  The phrase “unsatisfied remuneration regime” should better read as “unsatisfactory remuneration regime” | Thank you very much for your comment, we revised all the parts as you suggested by using suggested words/ expressions: students’ safety, non-academic work, inadequate eating and resting schedule at school, unsatisfactory remuneration regime |
| Page 7  Line 141: the word is “**administered”**  and not “administrated”  Line 144: The text should read “the data were” not “the data was”  Line 156: the text “ Decriptive data were presented” seems incomplete. Authors should describe how the data were presented. | Thank you very much for your comment, we revised all the parts as you suggested:   - Correct spelling and grammar - Add more detail in line 156. |
| Page 8  Line 170: The text should read “The final analysis of data” not “The final analysis of this paper”.  Line 173: The figure in bracket (85.7%) should come immediately after the word “most’ (Most [85.7%] respondents…) | Thank you very much for your comment, we revised all the parts as you suggested. |
| Page 9  Line 178: should read “prevalence of anxiety symptoms among the participants” not “prevalence of participants…” | Thank you very much for your comment, we revised the part as you suggested |
| Page 10  Table 2: it is not clear why the percentage figures presented, do not sum up to 100. Again, the authors did not indicate anywhere in the manuscript that the analysis presented involved only the participants who showed anxiety symptoms.  The authors need to also correct “Be criticized” and “Be disciplined” in the table. | Thank you very much for your comment. The % in table 2 did not add up to 100% because they were calculated separately for corresponding reference groups (exposure in 2x2 table). For example, comparing the prevalence of anxiety in the group with no intention to leave job (39.9%) with that in the group with unsure intention (53.3%)  We corrected “Be criticized” and “Be disciplined” in the table |
| Page 14  Line 253: The text should read “Parent-**teacher** collaboration plays a significant role in **students’** learning…” | Thank you very much for your comment, we revised the part as you suggested |
| Page 15  The authors have explanations for the high prevalence of anxiety in Malasia and Egypt, but has very little to say about the possible causes of the high prevavalence of anxiety in Vietnam. I suggest the authors discuss their findings more. “What might be the possible cause/causes of teachers’ anxiety? What are the likely consequences of teachers’ anxiety on the individual teachers, school children, and Vietnam society at large. | Thank you very much for your comment, we revised this part as you suggested by adding more explanation on the high prevalence of anxiety among teachers in Vietnam |
| Lines 273-274: The authors have still not discussed the finding on the relationship between teachers’ anxiety and intention to leave. The authors simply wrote that “a significant association was found between anxiety and intention to leave.” But nothing was said about the direction of the associaton. I expect the authors to further explain that teachers who had the intention to quit the job showed higher odds of anxiety symptoms than those who had no intention to leave the job.  Again, the authors wrote that their “finding imply that psychological interventions improve teachers’ mental health and help them stay with their current job.” However, I do not think that is the major implication of that finding. I think a major implication should be that “when teachers experience increased fear and worry about their work situations, they might lack the enthusiasm for the work and therefore, might want to quit the job. Conversely, teachers who already intend to leave the job, might experience increased anxiety as they handle the strain and stress associated with teaching young people.” | Thank you very much for your comment, we revised this part as you suggested by adding more explanation the association between anxiety symptoms and intention to leave and its implications. |
| Page 16  Lines 275 and 276: The text is a recommendation for future study. It should be taken to the last part of the conclusion section.  I believe these additional improvements will be helpful to the authors. | Thank you very much for your comment, we revised this part as you suggested by moving the sentence to the conclusion section. |
| reviewer 2:  Suggestions for the authors: |  |
| 1. In the abstract, you mention that one of the main findings is the lack of cooperation with colleagues, that is, with other teachers. However, in the body of the text and in the table in the results section, you state that it pertains to a lack of cooperation with parents. Please clarify this point. | Thank you very much for your comment, we checked the manuscript to ensure the consistency and revised the abstract |
| 2. In the introduction, the authors should specify what is the novelty element compared to previous studies, in order to stress which literature gap the document intends to address. | Thank you very much for your comment. We stated in the introduction that although teachers’ anxiety has been studied elsewhere, scant evidence is found in Vietnam. Among common mental disorders in teachers, stress and depression are more popular topics of research. Therefore, this paper aims to enrich the evidence on teachers’ anxiety in Vietnam as the base for appropirate and effective interventions. |
| 3. Line 66 and line 70 of the authors mention two studies in quotation marks, but do not provide the authors of the study. If it is a self-citation, please specify and cite appropriately (Author, year). | Thank you very much for your comment, this paper used data from another nationwide survey conducted by the Vietnam Education Union in 2020. We revised this part, remove the quote marks, and cited the reference. |
| 4. The authors used the DASS scale which measures anxiety, depression, and stress. However, the authors only present results related to anxiety. Was only this subscale used? Are there significant OR values for depression and stress as well? Why was this choice made? Please clarify and specify the rationale. | Thank you very much for your comment. The paper extracted data from a large survey and the authors only had permisison on anxiety data. The absence of stress and depresison data were discussed in the limitation section. |
| 5. In the methods, it may be helpful to specify the inclusion and exclusion criteria for teachers. | Thank you very much for your comment, we added more inforamtion on the inclusion and exclusion criteria for teachers. |
| 6. Certain variables, such as gender, were not taken into account in the design. There is a significant body of literature demonstrating that women, including teachers, typically have higher levels of anxiety, stress, and depression. | Thank you very much for your comment, we added more discussion about the gender difference, and one limitation that this study lacked gender information. |
| 7. Additionally, the difference between primary school teachers and secondary school teachers was not considered. They may face different challenges and have different levels of anxiety and stress. However, the comparative data, which could have been briefly addressed, are completely missing. | Thank you very much for your comment, we provided number of primary and secondary teachers in Table 1. Factors associated with anxiety status were examined separately for 2 groups of teachers in Table 2. |
| 8. The intention to leave the job is an alarm bell for work-related stress and could be an indicator of burnout. At this point, anxiety could be a response to the stressful situation, but unfortunately, this is addressed somewhat vaguely. I recommend that the authors better specify this in the discussions and conclusion, as it could provide a starting point for future studies. | Thank you very much for your comment, we revised this part as you comment and add more recommendation for future studies. |
| 9. It would be important to further investigate the specific situation that is being framed. What changes have teachers had to face in 2020? What specific educational challenges did Vietnam have to face during the pandemic? | Thank you very much for your comment, more details on the situation during 2020 were provided in the introduction and discussion. |
| In general, I believe the paper has potential. However, certain aspects are neglected in the way they are presented, making the work less detailed. I believe that adding details regarding the theoretical gap, sampling methodology, sample choice, possible connection with burnout, specific challenges of the country in question, would significantly enhance the clarity of the importance of this study that needs improvement.  In this sense, I hope to have contributed to highlighting aspects that can be strengthened in your work, and I wish you good luck! | Thank you very much for your valuable comments. |
