# Supplementary material for: The prevalence of anxiety and related factors among primary and secondary school teachers in Hanoi, Vietnam, during the COVID-19 pandemic in 2020
Source: PLOS Glob Public Health. 2024 Feb 28;4(2):e0002932. doi: 10.1371/journal.pgph.0002932 (PMC10901312; doi:10.1371/journal.pgph.0002932)
Supplement: Attachment — Submitted filename: Reviewers comment PGPH-D-23-00934_R2.pdf.docx [file pgph.0002932.s008.docx]

**Reviewer’s comment on manuscript PGPH-D-23-00934_R2.pdf**

The authors have greatly improved the manuscript. It is significantly better than the previous manuscript. However, I believe that addressing these few issues will bring out the best of the manuscript.

1. Page 6, Line 131 – it should be “students’ safety” (indicating plural) and not “student’s safety” (which refers to singular student).
2. Page 7

Line 144- there should be an article ‘the’ before the word “administered”.

3. Line 157

The authors wrote “Descriptive data were presented in count and percentage for category data and/or mean, standard deviation (SD), and min/max for age.”

I suggest the authors write “Descriptive data were presented in counts and percentages for categorical data while the age variable was also presented as mean, standard deviation, and minimum/maximum.”

In general, I suggest the authors to seek professional assistance for better use of singular and plural verbs/nouns and other grammar issues in their work.
