# Supplementary material for: The prevalence of anxiety and related factors among primary and secondary school teachers in Hanoi, Vietnam, during the COVID-19 pandemic in 2020
Source: PLOS Glob Public Health. 2024 Feb 28;4(2):e0002932. doi: 10.1371/journal.pgph.0002932 (PMC10901312; doi:10.1371/journal.pgph.0002932)
Supplement: Attachment — Submitted filename: Comments to the Editor PLOS.docx [file pgph.0002932.s009.docx]

**Reviewer’s comment to editor**

**Manuscript: PGPH-D-23-00934_R2.pdf**

Thank you for the opportunity to review the revised copy of this manuscript. The authors have improved the manuscript. However, I suggest the authors to seek professional assistance to proofread their manuscript for better use of singular and plural verbs/nouns and other grammar issues in their work.
